# Study protocol for an individually randomized control trial for India's first roleplay-based mobile game for reproductive health for adolescent girls

**DOI:** 10.1186/s12978-023-01665-1

**Published:** 2023-09-14

**Authors:** Ananya Saha, Anvita Dixit, Lalita Shankar, Madhusudana Battala, Nizamuddin Khan, Niranjan Saggurti, Kavita Ayyagari, Aparna Raj, Susan Howard

**Affiliations:** 1 Population Council Consulting Pvt Ltd, New Delhi, India; 2 Howard Delafield International, Washington, DC USA; 3 Faculty of Health Sciences, University of Ottawa, Ottawa, ON Canada; 4 School of Integrative Studies, College of Humanities and Social Studies, George Mason University, Fairfax, VA USA; 5 Population Council, New Delhi, India

**Keywords:** Outcome evaluation, RCT, Encouragement design, Adolescents, Mobile game, Digital, Sexual reproductive health

## Abstract

**Background:**

Go Nisha Go™ (GNG), is a mobile game combining behavioural science, human-centric design, game-based learning, and interactive storytelling. The model uses a direct-to-consumer (DTC) approach to deliver information, products, services, interactive learning, and agency-building experiences directly to girls. The game’s five episodes focus on issues of menstrual health management, fertility awareness, consent, contraception, and negotiation for delay of marriage and career. The game’s effectiveness on indicators linked to these issues will be measured using an encouragement design in a randomized controlled trial (RCT).

**Methods:**

A two-arm RCT will be conducted in three cities in India: Patna, Jaipur, and Delhi-NCR. The first arm is the treatment (encouragement) arm (n = 975) where the participants will be encouraged to download and play the game, and the second arm (n = 975) where the participants will not receive any nudges/encouragement to play the game. They may or may not have access to the game. After the baseline recruitment, participants will be randomly assigned to these two arms across the three locations. Participants of the treatment/encouragement arm will receive continuous support as part of the encouragement design to adopt, install the game from the Google Play Store at no cost, and play all levels on their Android devices. The encouragement activity will continue for ten weeks, during which participants will receive creative messages via weekly phone calls and WhatsApp messages. We will conduct the follow-up survey with all the participants (n = 1950) from the baseline survey after ten weeks of exposure. We will conduct 60 in-depth qualitative interviews (20 at each location) with a sub-sample of the participants from the encouragement arm to augment the quantitative surveys.

**Discussion:**

Following pre-testing of survey tools for feasibility of methodologies, we will recruit participants, randomize, collect baseline data, execute the encouragement design, and conduct the follow-up survey with eligible adolescents as written in the study protocol. Our study will add insights for the implementation of an encouragement design in RCTs with adolescent girls in the spectrum of game-based learning on sexual and reproductive health in India. Our study will provide evidence to support the outcome evaluation of the digital mobile game app, GNG. To our knowledge this is the first ever outcome evaluation study for a game-based application, and this study is expected to facilitate scalability of a direct-to-consumer approach to improve adolescent sexual and reproductive health outcomes in India.

*Trial registration number:* ctri.nic.in: CTRI/2023/03/050447.

## Plain language summary

Our paper describes implementation of a study protocol for an outcome evaluation of a mobile game app called Go Nisha Go™, produced by the Game of Choice, Not Chance™ project, funded by USAID. Consenting adolescent girls, aged 15–19, from three cities in India will be enrolled to participate in an encouragement design led RCT. Girls will be randomly assigned to either, a) a treatment (encouragement) arm where they will be nudged to play the game for ten weeks, or b) a control arm where participants will not be provided any encouragement to download or play the game. The study will be evaluated using surveys at baseline and follow-up. The findings from this study will support the measurement of effectiveness of the digital intervention and facilitate scalability of a direct-to-consumer approach, using a game-based application to improve adolescent sexual and reproductive health outcomes in India.

## Background

India is home to 253 million adolescents, where every fifth person is between 10 to 19 years [1]. Adolescent girls are facing a range of social, economic, and structural barriers including receiving adequate information on sexual and reproductive health (SRH), menstrual health and hygiene, fertility awareness, and the self-efficacy to seek health services and make choices for themselves. Fifty percent of girls aged 15 to 19 do not use menstrual hygiene products [2]. With limited access to requisite knowledge about menstrual hygiene, methods of contraception, self-care, and well-being, adolescent girls are at high risk of experiencing adverse health and social outcomes such as low educational attainment, lack of career aspirations, forced/early marriages, unintended pregnancy, and gender-based violence [3].

Interventions designed to minimize risks to adolescents include counselling through public health service providers, peer education, and community-based immersion by social development groups. Novel interventions that apply game-based learning to digital media such as mobile phone apps could increase motivation, engagement, and overall sustainability of health behaviours [4]. More recently, interventions that have incorporated serious games [5], through simulation and interactive methods, have gained significant attention from academicians, policymakers, and practitioners. The effectiveness of the next generation of games may be enhanced by building on behavioural change and educational gaming literature (e.g., using role-play and simulation game formats, individual tailoring, offering adaptation in the difficulty of the challenge, and the amount and timing of feedback), as well as human-centric design principles that follow user preferences [6].

Evaluation of a mobile game intervention to disseminate health-related knowledge and information and understanding use of game-based learning approaches to promote SRH, particularly to adolescents in low-income settings, has been limited. Generating evidence to examine causal impact of mobile games on adolescent SRH behaviour, longer-term health outcomes, self-efficacy and autonomy, and more expansive measures of well-being, is essential. Rigorous evaluations of game effectiveness, longer-term follow-up, and using measures of behaviour rather than merely their determinants, is warranted [7].

### Go Nisha Go mobile game

Go Nisha Go™ (GNG) is a role-play, simulation game [8], mobile app that follows the life of a young Delhi-based protagonist, Nisha, as she embarks on a journey of self-discovery and agency. Played across five levels, the game uses an aspirational [9] travel theme that has Nisha visiting the Indian cities of Sikkim, Mumbai, Goa, and Hyderabad, while meeting inspirational women (a scientist, an influencer, a nurse, and a police officer), as part of her internship for a web series production. Hindi is the spoken language of the game and Hinglish (Hindi language written in Latin script) is the script of the game. Thus, the interaction and communication with the game is made effortless [6]. Each level focuses on specific game-based objectives related to menstrual health, fertility awareness, contraception, and consent, scaffolded on outcomes related to negotiation, relationship obligation, health knowledge, and confidence. The game is designed to increase knowledge incrementally (and subsequently the confidence and efficacy to make better choices) through strategically placed ‘nudges’ in the gameplay, trade-offs/rewards, minigames, and direct access to resources such as educational videos and a chatbot. The game does not collect any Personally Identifiable Data (PID), thus protecting players’ privacy.

### Evaluation framework and theory of change (TOC)

The Go Nisha Go (GNG) evaluation framework [10] is underpinned in theoretical frameworks such as the Integrated Model of Behaviour Change [11] and the Fogg Behaviour model [12, 13]. These theoretical models define the innovative intervention used to address knowledge, attitude, self-efficacy, and practice indicators for SRH among girls aged 15 to 19 years.

The TOC [8] informs a robust results framework with the following action-oriented Intermediate Results (IR):

- IR1: Improved knowledge about SRH care including menstruation, menstrual hygiene, fertile period, and SRH/menstrual management products.
- IR2: Improved attitudes, confidence, and decision support for managing self-care, family planning, negotiating consensual sex and contraceptive use, and accessing/using DTC products, information, and care.
- IR3: Improved assertion of self-identity through DTC linkages to career, safety, and personal well-being resources, and improved self-efficacy through linkages (access) to SRH products, information, and care.

In this paper, we describe the study protocol for the implementation of an outcome evaluation (OE) that measures (a) increase of knowledge of select SRH indicators, including fertility awareness, comprehensive contraceptive knowledge among adolescent girls; (b) improved access to SRH information, products, and services via DTC links within the game; and (c) intended behaviour change actions that increase agency and confidence among adolescent girls for successful SRH outcomes.

## Methods

This study protocol paper is aligned to SPIRIT 2013 guidelines [14]. The study is designed as a two-armed individual Randomized Control Trial (RCT) comprising a treatment (encouragement) arm and a control arm.

### Study setting

The study will be conducted in three Indian peri-urban areas of three states—Patna in Bihar, Jaipur in Rajasthan, and Delhi NCR (National Capital Region).

### Selection/eligibility criteria

Eligible participants are unmarried adolescent girls aged 15 to 19 years, living in Patna-Bihar, Jaipur-Rajasthan, and Delhi NCR, able to read Hinglish (Hindi and English hybrid in Latin Script), and who have access to an Android-based device with internet. We will note the demographics of participants who refused to be part of the study, which will be analyzed for participation bias.

### Pretesting of methods and learnings

In preparation for the RCT, we pre-tested the study protocol for feasibility of methodology and implementation guidelines, where we recruited 270 girls to determine which of three modes (in-person surveys, online self-administered surveys, and telephonic interviews) of recruitment could be finalized. For in-person surveys we recruited 120 girls: (n = 40) in Patna, (n = 40) in Jaipur and in (n = 40) Delhi NCR. Similarly, for the telephonic surveys and online surveys respectively, 60 girls each were recruited in total: Patna (n = 20), Jaipur (n = 20), and Delhi NCR (n = 20). We also tested a qualitative tool through in-depth interviews (IDI) with 30 girls in the three cities of Patna (n = 10), Jaipur (n = 10), and Delhi NCR (n = 10).

All participants were recruited from an eligible list of participants after a household survey. The interviews were conducted in a confidential and comfortable space in the participant’s household/community after taking consent for participation in the study.

The pretesting aided in examining the feasibility of the methods and determined that the response rate of in-person interviews (face-to-face) was higher than the other two modes. Refusal to take interviews over the telephone was considerably high. In the online self-administered interviews, many girls opted out, saying they were not comfortable with sharing personal information online. We also found that school examination times interfered with the recruitment of participants, and therefore made plans to conduct the encouragement study after exams. We also pretested creative messages for the encouragement and determined that additional creative messages were needed to motivate participants to not only download the game, but also play all five levels. ﻿The two-armed RCT will now be conducted only through in-person mode of recruitment after exams.

Additionally, to ensure comparison with outcome evaluation data and in-game metrics, we will now ask recruited participants to download a unique six-digit profile code that will be generated at start of gameplay. The profile code from the participant will determine how data from the OE can be matched with the in-game choices of the same player.

### RCT study

#### Sample size

The study proposes recruiting 1,950 participants from three sites: 650 girls each from Patna, Jaipur, and Delhi NCR for in-person interviews. In addition, 60 girls (n = 20 each from Patna, Jaipur, and Delhi NCR) will be recruited purposively from the encouragement arm of 975 participants for qualitative in-depth interviews. During the encouragement design activity, half of the participants (i.e., 325 girls each from Patna, Jaipur, and Delhi NCR) will receive nudges/encouragement to play the game.

#### Recruitment

Eligible participants of the study will be identified by trained surveyors in-person through the eligibility screening process and household listing. Following this, a sampling frame with potentially eligible respondents and their parents (for minor eligible adolescent respondents who are below 18 years) will be approached by the field team for consent and recruitment into the study. A Field Coordinator and Supervisor will monitor this process including the sketching of Primary Sampling Units (PSU) to ensure that mapping and recruitment is done ethically as per standard protocols. The final recruitment of participants will be selected from a household listing in the eligible criteria: unmarried, adolescent girls ages 15 to 19, having their own or shared mobile device with internet access.

#### Randomization

**Fig. 1:**
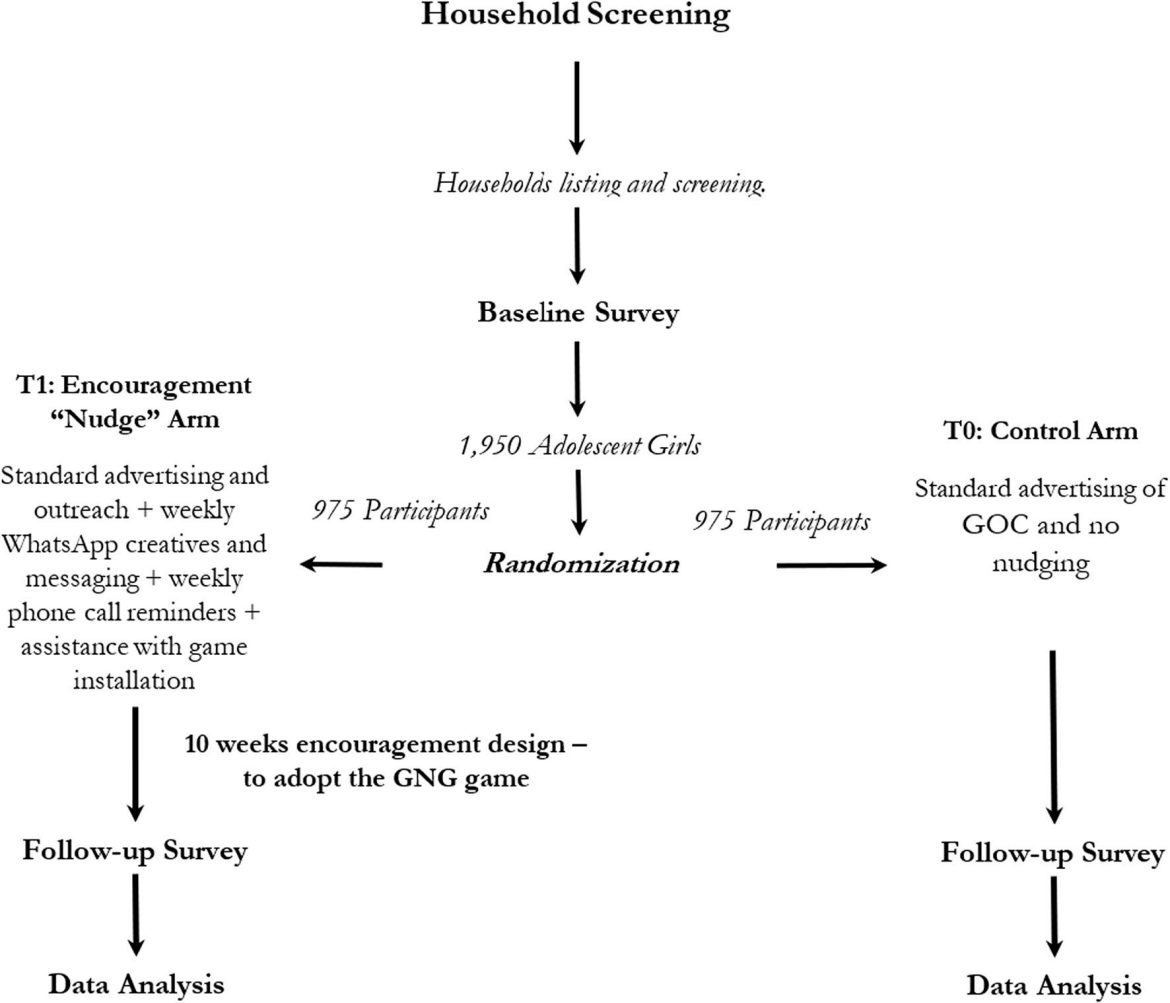
Outcome Evaluation—Randomized Control Trial Study Design

For implementation of the RCT we will prepare the eligible list of participants after household screening, followed by individually randomizing the total participants into control and intervention groups [15]. Randomized participants in the treatment arm will get exposure to the GNG game as part of the encouragement design, while the participants randomly assigned to the control arm will not get any additional encouragement to download or play the game. (See Fig. 1) Researchers will not be blinded to the treatment condition assigned to individual participants [16].

#### Encouragement design

Ten weeks of encouragement activity will be designed for participants to download and play the game with continuous nudges in the form of phone calls and WhatsApp messages (see Table 1), as well as follow-up scripted phone calls from the program facilitators.

**Table 1:**
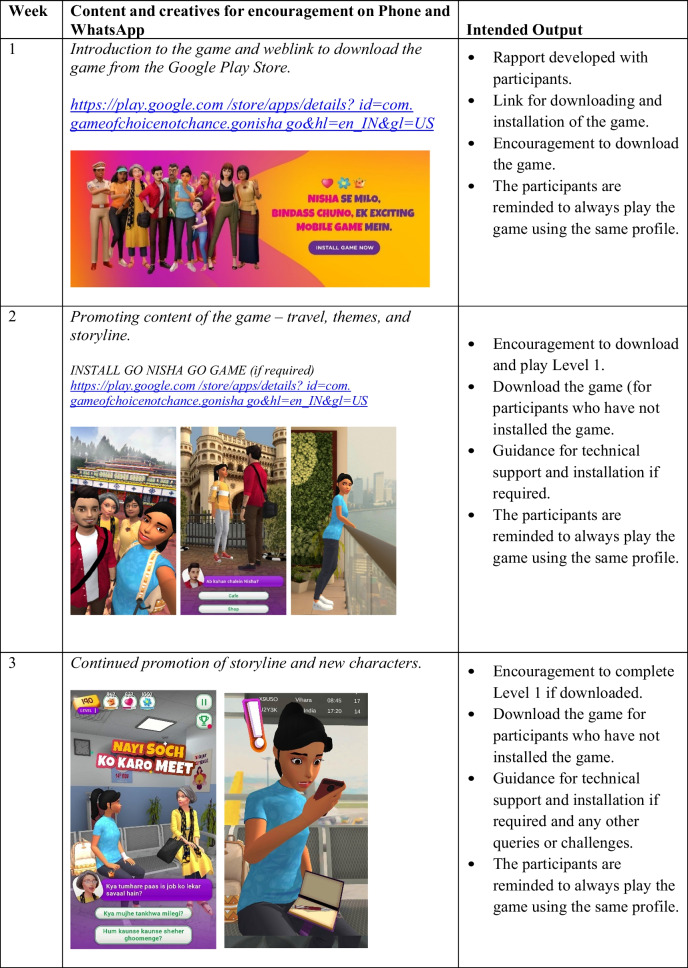

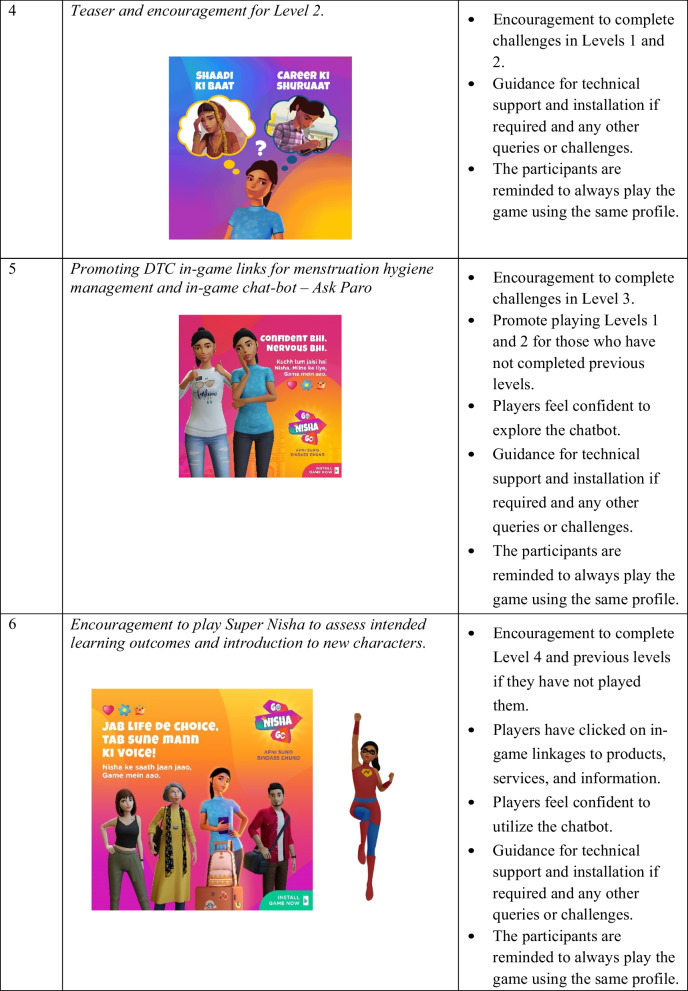

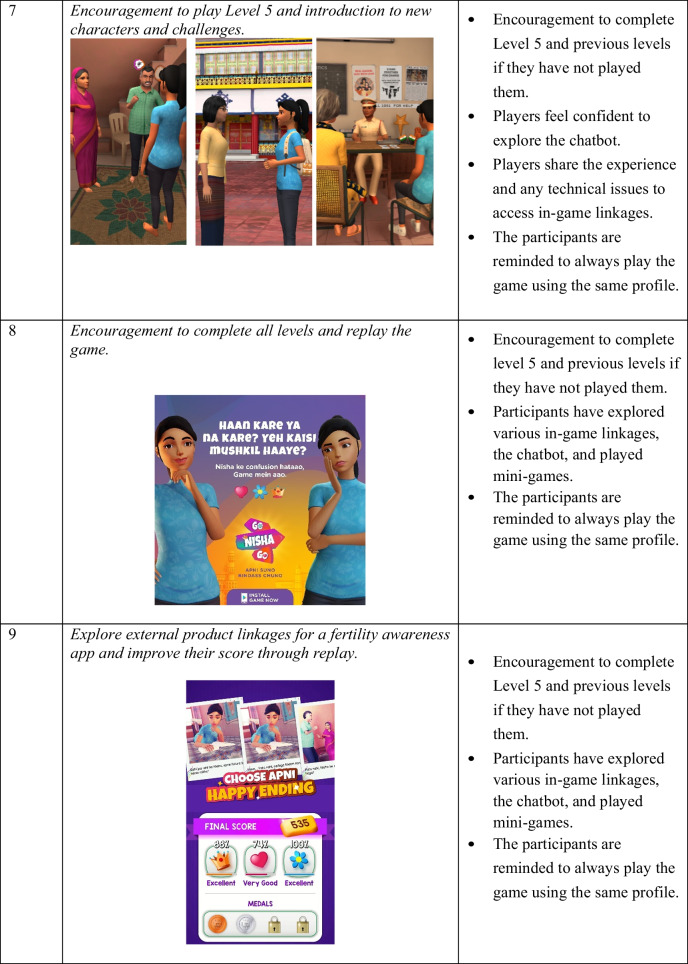

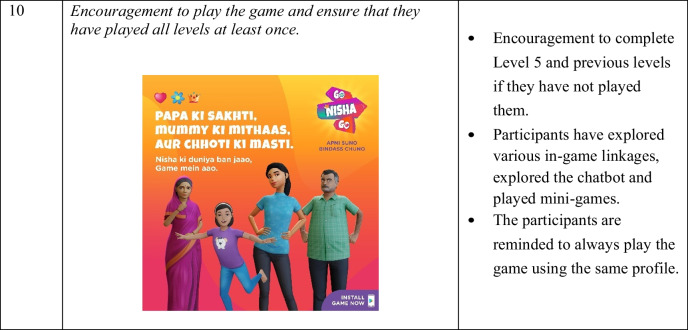
Encouragement design for a ten-week intervention for GNG evaluation

#### Timepoint for tracking encouragements for participants

We will follow up with the participants by using a tracking sheet. Please see Table 2 for the schedule of enrollment,  implementation of encouragement, and assessments.

**Table 2:** Schedule of enrolment, intervention, and assessment

Timepoint**	Study period*			
	Enrollment	Allocation	Post-allocation	
	−t_1_	0	t_1_	t_2_
Enrolment				
Eligibility screen	X			
Informed consent	X		X	X
Allocation		X		
Interventions: [Intervention: GNG game encouragement design]			↔	﻿↔
Control: No Encouragement				
Assessments: Baseline variables – indicators (see Table 3)			X	
Menstrual hygiene			X	X
Contraception			X	X
Agency (decision-making, negotiation, and consent)			X	X

*Recommended content can be displayed using various schematic formats. See SPIRIT 2013 Explanation and Elaboration for examples from protocols

**List specific timepoints in this row

## Baseline and follow-up survey tools

### Quantitative survey

After completing the household listing exercise, the eligible participants will be identified and listed with the help of a screening tool. The final list of respondents will be prepared after de-identifying the individual participants into unique identification respondent IDs. For the main phase RCT study, we will recruit adolescent girls based on the previously mentioned inclusion criteria. The final list will be considered the sampling frame for the recruitment of baseline data collection. Once the sampling frame is developed, the eligible adolescent girls will be approached for data collection of in-person surveys.

The survey tool was prepared and tested in the pretesting of the method. There are eight sections in the baseline questionnaire and nine for the endline. (See Table 3).

**Table 3:** Sections in the survey tool

Section	Theme	Description
I A	Household Characteristics	Details of household features, including understanding socio economic level of the household related to household goods/financial condition
I B	Individual Characteristics	Background information on age, siblings, parents, education, livelihood, friends, and career aspirations
II	Media and Technology	Phone and media consumption patterns
III	Health Services Utilization	Availability, accessibility, and preferences for healthcare services
IV	Menstrual Health and Fertility Awareness	SRH information/services received, perception, and attitude towards MHM
V	Empowerment and Aspirations	Understanding of personal aspirations, knowledge/attitudes about career aspirations, preparation for job/career, and financial independence
VI	Negotiation and Consent	Knowledge of attitudes and perceptions towards personal agency, consent in relationships, the legal age of marriage, and consent for sex
VII	Male and Female Relationships	Age-appropriate questions on types of relationships (platonic/physical), attitudes towards dating, perception of acceptable activities in a relationship
VIII	Contraception	Knowledge of methods of contraception, accessibility, and utilization
IX Only for endline survey	Knowledge, Attitude, and Practices: Go Nisha Go	Knowledge of GNG game, levels played, links accessed, product and brand recall, gameplay, and intention for behaviour change and measurement of confidence for negotiation with partner/parents

#### Ethical consideration and consent/assent process

Prior to administering the survey, respondents will be asked for their consent, using standard format forms. Surveys will be administered in a private room/safe space in the participant’s residence/community and will last approximately 45–60 mins (based on our pre-testing experience). To protect participant privacy and ensure confidentiality, all Personally Identifiable Data (PID) will be appropriately stored and secured in accordance with the data security measures. All survey forms will only contain a unique ID code generated for the study.

Due to the sensitive nature of questions being asked, participants may feel personal discomfort, fatigue, or embarrassment during survey administration. Participants will be reminded that their participation is completely voluntary. They do not have to answer any question that they do not feel comfortable answering, and they can decide to end their participation in the study at any time. We do not anticipate respondents to be subjected to more than a minimal risk of discomfort from participating in this study. Additionally, our secure storage, encryption, and coding of all data, particularly PID, serves to minimize any risk of breaching confidentiality and compliance with local data privacy law.

Study participants will be able to access the game for free directly from their own Google Play Store account. Once downloaded the GNG game has clear terms of service that need to be accepted by the player, including preconditions for downloading the game if the person is under the age of 15. Participants who do not own their phones or share their phones with family members, may run the risk of backlash from male members/parents who may have reservations about access to SRH information among adolescent girls. In this regard, we will recruit credible community members and peers who are tasked to brief parents about the goal and objectives of the game, and the content will be aligned with the curriculum outlined in the government program for adolescents.

#### Confidentiality

All steps to ensure that participants’ information will be kept confidential by trained study staff to ensure that collected information will be secured and will not be shared with others. In the event that we find out through the study that participants are at risk of harm or there is a risk of harm to others, we will need to break confidentiality in order to provide an active referral for services, and/or report this risk to the relevant authorities (Indian Sigma Institutional Review Board (IRB) and the international Population Council IRB). We will ensure that participants understand that they can refuse to answer any question or stop participating in the study at any time.

#### Data security

To effectively monitor participants over the study period, identifiable data on participants’ demographic backgrounds, and personal contact information (household addresses, phone numbers, and e-mails) will be collected as part of the in-person survey. The data collected from in-person surveys will be administered in an electronic Computer Assisted in-Person Interview (CAPI) format, using the CSPro survey management system utilized by the U.S. Census Bureau [17]. Electronic survey data will be collected by interviewers on Android-based tablets, and data will be securely transferred from the Android tablets onto an Amazon Web Services (AWS) supported, secure, cloud server at the end of each working day. All Android tablets will be used for data collection only, and tablets’ settings will be adjusted so that field staff is blocked from accessing applications that are not applicable for data collection (e.g., internet browsing, social media, email). The AWS cloud server will be HIPAA-compliant and will meet all the necessary security requirements for storing Level 4 identifiable data. Once the data has been securely transferred to the cloud server, the survey record on the Android tablet will be immediately erased.

#### Informed consent process

Participants who are under the age of 18 are minors who cannot voluntarily provide consent for themselves, and therefore, will require parental consent with participant assent to participate in the study. All consent and assent processes with parents and adolescents will be administered in person, following household listing and recruitment of eligible households. Some members of this population may be subject to the influence of others, particularly parents and other elder members of the household, due to power relationships internal to the household and/or the community.

### Follow-up survey

Following the ten-week intervention period/encouragement, participants will be assessed through the administration of the follow-up survey. All procedures related to informed consent, confidentially, data privacy, and risk mitigation will be followed, as will been done in the baseline. Resurveying participants in this manner will enable us to create a panel of individual girls, in which each girl is observed over two time points. In the endline survey, we will collect data using the same survey tool, with an additional section on gameplay and game-related elements, including brand recall of in-game links to products and services.

#### Qualitative in-depth interviews

After the follow-up survey, we will administer in-depth interviews (IDI) with a sample of 60 adolescent girls from the three study sites (20 girls per site). The purpose of the qualitative interviews is to complement the quantitative data analysis, to improve our understanding of adolescents’ experiences with the GNG game, such as: (1) Gain better understanding of the SRH environment for adolescents, including established gender norms, expectations around relationships and intimacy between adolescent girls and boys, and attitudes towards sex, marriage, girl’s health—including MHM, fertility awareness, contraception, and pregnancy; (2) Assess the level of access and availability of menstrual and contraceptive knowledge, information, products, and services through the DTC approach; and (3) Evaluate the agency and level of confidence for intended behaviours related to improved SRH outcomes, such as decision making and consent, and negotiation for delaying early marriage and pregnancy.

As with the quantitative surveys, we will explain the purpose of the interviews to participants of the qualitative survey, taking consent/assent before starting the interview. Girls will be selected for the personal IDIs in accordance with the inclusion and exclusion criteria. Interviews and debriefing activities will be administered in private rooms, or outdoor areas where privacy can be assured, and will last approximately one hour per participant. Interviews will be conducted in Hindi. Some interview questions will be open-ended, although most of the interview will follow the interview guide, which will help to ensure that key questions will be asked, and key domains will be addressed.

The in-depth interviews will be conducted by trained qualitative female research investigators between the ages of 21 to 30 years. All interviews with the participants will be digitally taped by the interviewers using audio recorders. If the participants refuse this process, we will administer the note-keeping process during the interview. After completion of the interviews, the investigator will save the audio recording in the protected computer system﻿, CAPI, assigned by the study team. The interview notes will be translated into Hindi and into English, then labelled and stored in the same interview folders on the computer. After completion of each interview, the audio recordings will be translated from Hindi to English, transcribed, and labelled. In case of non-availability of the audio recording, the interviewer will make a copy of notes from the interview, label, and save it in txt. format on the computer. All PID will be removed from interview forms and a unique ID number will be assigned. Coded forms will be kept separate from the code list to maintain confidentiality.

All linking files will be stored separately in a locked cabinet at the Data Collection site. Audio recordings of the interviews and related data will also be stored separately from the transcripts of the interviews. Both recordings and transcripts will be stored in a locked facility. All study members will be trained in maintaining participant confidentiality.

#### Outcomes measures

The baseline and follow-up survey tools are created with the previously tested tools from standard studies such as the UDAYA study (Understanding the lives of adolescents and young adults) [3], National Family Health Survey (NFHS) [18], Comprehensive National Nutrition Survey (CNNS) [19], Living Standards Measurement Study [20], and other national surveys in India [21]. The primary outcome of the evaluation is to measure improvement in the existing knowledge of MHM, contraception, and increased in agency for adolescent girls making informed decisions and choices in their lives (See Table 4). Several other secondary outcomes measuring intended behaviour change and agency with respect to improvement in decision making, choice of career, negotiation in relationships, and assertion of self-identity will also be evaluated.

**Table 4:** List of selected outcome indicators for the study

Outcome indicators	Contents of measurement
Menstrual health management	Percentage of girls who have: - Received any information about MHM - Awareness about sanitary napkins - Awareness about different menstrual hygiene products - Obtained menstrual products alone - Practiced tracking their menstruation with comprehensive understanding of fertility awareness - Experienced trouble in obtaining menstrual hygiene products - Bought menstrual hygiene products online
Contraception	Percentage of girls who have: - Awareness about modern contraceptive methods - Comprehensive knowledge about Oral contraception pills - Comprehensive knowledge about Emergency contraception pills - Comprehensive knowledge about condoms - Comprehensive knowledge about IUD - Comprehensive knowledge about injectables - Practiced refusing sex due to non-availability of contraception - Awareness about condom’s use for prevention of pregnancy and/or infections
Agency (decision making, negotiation, and consent)	Percentage of girls who/ have: - Gone to college on their own/alone, - Believe that “girls should make decisions for themselves”, - Believe that “girls can wear what they want”, - Believe that “girls should manage their own money”, - Believe that “girls should be financially independent”, - Believe that girls can talk to their parents when they were asked to marry a person whom they do not want to marry, - Feel confident to say no to a sexual act, - Feel confident to use condoms when her boyfriend is not willing to, - Think their boyfriend should always take consent for sex and refuse sex due to non-availability of contraception

#### Data analysis plan

Analyses will test whether participants who played the game (treatment arm) relative to control participants who have not received any encouragement to play the game:

- Improved knowledge about SRH care, including menstruation, menstrual hygiene, fertility awareness, and SRH/menstrual management products.
- Improved attitudes, confidence, and decision support for managing her self-care, SRH, negotiating consent and contraceptive use, and accessing/using DTC information, products, and care.
- Improved assertion of self-identity through DTC linkages to career, safety, and personal well-being resources, and improved self-efficacy.

Analysis of the quantitative study data will be conducted using STATA and SPSS where appropriate. Descriptive analysis will be performed for all variables, and unadjusted comparisons between experimental and control arms will be conducted. Descriptive statistics will be performed, including frequencies, means, and standard deviations. In addition, chi-square tests and t-tests will be used to examine associations in the data. A probability value of less than 0.05 will be considered statistically significant for all statistical tests conducted. Continuous variables will be tested for normality, and non-normal values will be categorised or transformed appropriately.

The following two analyses will be done to satisfy the objective of the study:

1. Univariate descriptive analysis such as frequency, percentages, the mean, and standard deviation will be computed for different background characteristics and knowledge, attitude, and practice (KAP) variables.
2. KAP variables will be analyzed using Z-test/t-test for encouragement and control arms in two points from the same respondent.
3. The net change in KAP and its significance will be calculated using difference-in-difference (DID) analysis. The DID analysis will be done using the following formula. (See Table 5)

**Table 5:** Difference-in-difference analysis pathway in RCT

	Experimental arm (s = 2)	Control arm (s = 1)	Difference (s)
Baseline (t = 1)	Y_12_	Y_11_	Y_21_ –Y_11_
Follow-up (t = 2)	Y_22_	Y_21_	Y_22_ –Y_12_
Difference (t)	Y_22_ –Y_21_	Y_12_ –Y_11_	Net change (DID) = (Y_22_ – Y_21_) – Y_12_ – Y_11_

The level of significance of DID will be tested using the below equation through regression analysis.

$${\text{Y }} = {\text{ a }} + {\text{ b}}_{{{\text{1s}}}} + {\text{ b}}_{{{\text{2t}}}} + {\text{ b}}_{{{\text{3s}}*}}$$

where.

Y = dependent/response variable.

a = constant.

s and t are study arm and time, respectively.

b_1_ and b_2_ are slopes for the study arm and time, respectively.

b_3_ = treatment effect.

s*t is a dummy variable indicating treatment status.

The significance level of the test will be measured using a p-value < 0.05 (95% level of significance as per the sample size estimation).

The primary comparison of the binary outcomes between intervention groups at baseline and follow-up will use mixed-effects longitudinal logistic regression, with random effects for the individual over time. The baseline will be included as a time point only. A categorical time effect will be used (profile model). A “difference-in-differences” analysis will compare the treatment arms. Statistically, this amounts to testing for an interaction between time and treatment arm in the longitudinal model. Since the treatment assignment is at random, this “unadjusted” analysis will provide a causal effect of the intervention. However, we will also conduct secondary analyses, adjusting for potentially relevant covariates (e.g., age, education, etc.), in addition to time and treatment arm, using backward model selection at the α = 0.15 threshold level. These secondary analyses may improve the precision (power) of estimating the treatment effect and reduce any bias due to random imbalances between arms.

The primary analyses will use an intent-to-treat approach and analyze all subjects according to a randomized group. For subjects missing the follow-up survey, we will impute data from the lower (worse) half of the distribution by the arm (conservative approach). We will also conduct dose analyses, in which we include only those subjects who complete the full intervention. Analyses will use data only for outcomes; (a) increased comprehensive contraceptive knowledge, (b) MHM (c) negotiation and self-efficacy. We will conduct exploratory analyses using outcomes.

#### Qualitative analysis

After the transcriptions are in place, the study team will import the text files in the qualitative data analysis and coding software – ATLAS.ti® [22]. The team plans to analyse these data with in-depth interviews, using the grounded theory approach implying the inductive and deductive method [16]. The researchers will try to understand the influence of emerging themes in the game intervention. We will dive deep into the data to find any interlinks between the current intervention themes of the game and emerging thematic areas during the interviews. The analysis will emphasize themes such as decision-making attitudes of adolescents, MHM, choice of contraception/contraception knowledge, and career aspirations. The approach to the analysis of these data is as follows:

1. Field immersion: The field investigators and principal investigators will read and reread the transcription and associated field notes from the sites to get familiarized with the data flow. This will help in developing a narrative and create memos to guide in broad theme development.
2. Thematic areas—The field investigators under the guidance of the principal investigators will develop a list of emergent key themes, seen across intervention areas.
3. Framework development—Our scientific investigator team will review and discuss the themes generated and their intersections, to create a framework for understanding the issues of focus. We may develop revisions to protocols or extensions of data collection to better gain more insight.

Codebook development- The senior scientist investigator will develop a codebook based on the framework themes, and a coding structure will be created to guide further data analysis. Coding and iterative analysis - the researchers will code and analyse all data using themes generated in steps 2–4. We will have one coder review and code each transcript. Coder will code separately, and codes will be analysed for intercoder reliability based on Cohen’s Kappa [23]. We will review coding discrepancies to reach consensus. New codes may emerge, and we may expand the codebook and conduct another analysis of the data.

Based on the findings, the framework will be refined to understand the objective of the gameplay to initiate conversation around menstruation, contraception, and decision making.

#### Data safety, monitoring, and protection for human subjects

We will develop our study protocol in accordance with the guidelines provided by the institutional review boards. We will strictly follow the ethical consideration to maintain encouragement of the study participants in the treatment arm. Regarding the data safety and monitoring of the study participants, we will focus on three major aspects: (a) assurance of no harm reaches the study participants to take part in the study, (b) assurance of data security—no PID is collected and shared to maintain the privacy of study participants (c) assurance to minimize risk for the commencement of adverse events. The study participants will be provided with the state’s helpline numbers and the community level organizations will be involved to safeguard participants.

Should there be an occurrence of any adverse events, the principal investigators of India will be informed within 72 h. The field investigators will be trained in handling situations at the community level, where maintenance of confidentiality and privacy is imperative to the study. The study team will continuously work on reducing risk and harm if caused during the study period. The principal investigator and senior researcher will diligently monitor and provide guidance to the field teams. As mentioned earlier, data collected will have no personal identifiers in the data. The data will be uploaded in AWS secure cloud server on a real time basis. All study computers used for descriptive analysis of the de-identified data will be password protected, and only study staff who are cleared to view the data will have the password. All study data and linking keys will be password protected at all times. All electronic files will be backed up nightly to minimise the likelihood of lost files or data. All electronic data, both on the AWS secure cloud server and on any study computers, will be encrypted and password protected. Identifiable hard-copy data, including signed consent forms, will be collected and stored in a locked cabinet in access-limited rooms at the office. The information key will only be accessible to the research team.

#### Ethics approval

The study has received the research ethical approval from the Indian Sigma Institutional Review Board (IRB) and the international Population Council IRB. Both IRBs have approved the research protocol design, consent forms, and conduct of the study in the three Indian cities – Patna, Rajasthan, and Delhi. The study team will inform the respective IRBs for any modification and update of the protocol for the trial registration, after agreement of all partners of the encouragement trial.

## Discussion

Most evaluations of mobile games for health or digital interventions assess cognitive, affective, behavioural, and biological functioning via self-reporting. Ideally, self-reports should be combined with rigorous measurements of observed health behaviours and outcomes to assess the impact of the game [24].

Moreover, rigorous evaluations of the causal impact of digital interventions are less convincing when control (comparison) groups are not well identified. We plan to implement a robust RCT-led encouragement design to evaluate the effectiveness of a digital mobile game app for selecting improved SRH outcomes. As the digital revolution expands with deeper market penetration of mobile phones, there is an opportunity to improve the accessibility and availability of resources for adolescents, so that their optimal health remains a key determinant of India’s overall health, mortality, morbidity, and population growth scenario [25].

To the best of our knowledge, the use of granular measurements, such as a scientifically backed RCT to measure mobile game outcomes, as against heuristic dashboards for demographically relevant content such as fertility awareness and contraception, is a first of its kind in India. We plan to complete the OE study by the end of 2023. The findings from the study are expected to demonstrate increased levels of knowledge, as well as change of intended behaviours related to MHM, contraceptive use, and negotiation with parent/partner for improved agency and decision making among adolescents. Additionally, we hope to provide evidence for accelerating scale of uptake and facilitating a new DTC digital mobile app that could provide access to key SRH information, products, and services to adolescent girls who value confidentiality, and would prefer to bypass traditional gatekeepers of health information and services.

## Data Availability

Not applicable.
